# Health-behavior intervention increases sedentary breaks in children aged 0–5 years: evidence from the Melbourne Infant Feeding, Activity and Nutrition Trial

**DOI:** 10.3389/fdgth.2026.1720371

**Published:** 2026-02-25

**Authors:** Luke Boerdijk, Kylie D. Hesketh, Carry M. Renders, Katherine Downing, Simone J. J. M. Verswijveren

**Affiliations:** 1 Institute for Physical Activity and Nutrition (IPAN), Deakin University, Geelong, VIC, Australia; 2 Department of Health Sciences, Faculty of Science, Amsterdam Public Health Research Institute, Vrije Universiteit Amsterdam, Amsterdam, Netherlands

**Keywords:** activity patterns, early childhood, physical activity, RCT - randomized controlled trial, sedentary behavior, movement behaviours

## Abstract

**Introduction:**

Early childhood physical activity and sedentary behavior influence long-term health, yet evidence on interventions targeting how these behaviors are accumulated – rather than just total time – is limited. This study examined the impact of a parent-focused early childhood obesity prevention intervention on preschoolers’ physical activity and sedentary behavior patterns.

**Methods:**

This study is a secondary analysis of data from the Melbourne Infant Feeding, Activity and Nutrition Trial [InFANT] clustered randomized control trial (2008–2013). Physical activity and sedentary behavior data were gathered using ActiGraph™ GT1M accelerometers. To capture insights beyond total time and examine accumulation patterns, the duration and frequency of sedentary bouts [≤100 counts per minute (cpm)], light-intensity physical activity (101–1,680 cpm) bouts, and moderate- to vigorous-intensity physical activity (≥1,681 cpm) bouts, each lasting ≥1 min, as well as the total number of sedentary breaks, were calculated at each time point. The ≥1 min cut-offs for bout durations were defined based on the medians observed in this sample (0.50 min for sedentary, 0.47 min for light-, 0.25 min for moderate- to vigorous-intensity physical activity). Multilevel regression analyses were fitted to examine intervention effects at 19 months (T3), and 3.5 (T4) and 5 (T5) years of age.

**Results:**

In total, 296, 144 and 140 participants had valid accelerometry data and were included in the analytical sample at T3, T4 and T5, respectively. The intervention had a significant and beneficial effect on the total amount of sedentary breaks at 3.5 (*β*[95%CI] = 10.9[2.98,18.91) and 5 years (*β*[95%CI] = 7.94[0.03,15.86). The intervention effects on all other outcomes were small and not significant.

**Discussion:**

Whilst only effects on sedentary breaks were observed, this study suggests that interventions may impact accumulation patterns in children under five.

**Trial registration:**

The Melbourne Infant Feeding, Activity and Nutrition Trial was registered with the International Standard Randomized Controlled Trial Number Register (ISRCTN81847050; 7/11/2007).

## Introduction

Approximately 1 in 4 children aged between 2 and 4 years experience overweight or obesity (1). Overweight and obesity in childhood are linked to a lower quality of life in both childhood and adulthood, leading to a significant burden on society (2–5). In 2015, 8.7% of the total burden of disease in Australia was attributed to overweight and obesity (1). To limit this burden of disease, it is important to investigate ways to lower the proportion of children with overweight and obesity.

It is well established that engaging in sufficient physical activity (PA) and limiting sedentary behavior (SB) reduces the risk of overweight and obesity in children under five (6–8). Current Australian guidelines for children aged ≤5 years focus primarily on total daily volumes of PA and screen time (9). However, these guidelines do not provide recommendations on *how* such behaviors should be accumulated across the day (9). This is an important omission, as growing evidence in school-aged children and adults indicates that health outcomes are influenced not only by the total amount of PA and SB, but also by their accumulation patterns (10–17). Understanding these patterns in early childhood is critical, as they may represent modifiable targets for interventions to optimize healthy growth and development.

Accumulation patterns are defined as the temporal structure of PA and SB accumulated over a specified period during the waking hours (18) and are typically operationalized as bouts and breaks in PA and sedentary time. Accumulation patterns have been shown to be independently associated with health outcomes. For example, an increase in the time spent in moderate- to- vigorous-intensity PA (MVPA) bouts [both short (<10 min) and long (> 10 min)] was inversely associated with having overweight, in adults, even when controlled for total MVPA (13, 15). The few studies that investigated intervention effects on accumulation patterns have mostly focused on school-aged children (19, 20). For example, Verswijveren et al. (19) found small and non-significant changes in accumulation patterns at 18 months post-intervention as a result of the Transform-Us! school- and home-based cluster randomized controlled trial. However, a follow-up study showed significant differences in the frequency of sedentary breaks between the intervention and control group at 30 months post-intervention (19, 20). Nevertheless, the current available evidence on accumulation patterns and whether they can be changed in children aged ≤5 years, is inconsistent and limited (21).

In early childhood, interventions targeting PA and SB, often to prevent obesity, have been delivered across a range of settings, including early childhood education and care [ECEC; (22)], family-based [e.g., (23)], healthcare [e.g., (24)], and community contexts, with digital delivery increasingly common (25). While ECEC-based interventions show promise, children often have time-limited exposure as they only spend a part of the week in and may move between care settings. A key strength of parent-focused interventions delivered early in life is the potential for sustained benefits, given the continuity of parental care across childhood. For example, the Preventing Overweight in Infancy trial (24) demonstrated sustained reductions in obesity prevalence among children exposed to a parent-focused intervention at 3.5 and 5 years of age, despite limited effects at intervention completion. Notably, however, many early-childhood interventions have focused on the total volume of PA or SB accumulated across the day. Far fewer have examined how these behaviors are distributed or accumulated across daily routines, particularly among children under five years of age. Such approaches could consider behaviors including screen time and physical restraint (e.g., prolonged times in prams instead of walking) in very young children. Parent-focused interventions delivered early in life may be especially well placed to shape daily routines and movement opportunities, and in turn influence these accumulation patterns.

Within this context, the Melbourne Infant Feeding, Activity and Nutrition Trial (InFANT) adopted a parent-focused approach to promote behaviors that prevent obesity, including increasing PA and decreasing SB. Informed by social cognitive theory and guided by an anticipatory guidance framework (26), the intervention aimed to equip parents with knowledge and strategies to promote healthy PA, SB, and dietary intake, aligned with their child's developmental stage. In early childhood, parents play a central role in shaping children's daily routines, activity opportunities, and behavioral norms (27). Accordingly, parent-focused interventions, such as the Melbourne InFANT program, may influence accumulation patterns through multiple pathways, including changes in parenting practices, parental role modelling, reinforcement of active play and reduced sedentary time, and restructuring of the home environment to support frequent movement and interruptions to prolonged sitting. While previous analyses have reported no significant effects of the Melbourne InFANT Program on total duration of PA or SB, aside from reductions in television viewing (28), it remains unclear whether the intervention influenced how PA and sedentary time were accumulated across the day. Therefore, the current study aims to examine the effect of the Melbourne InFANT program on accumulation patterns of PA and sedentary time.

## Materials and methods

### Study design

The Melbourne InFANT program was a cluster randomized controlled trial that aimed to prevent obesity and obesity-related behaviors in children aged 4–19 months, with follow-up at ages 3.5 and 5 years (June 2008 to February 2013). The Melbourne InFANT program employed anticipatory guidance (26), where healthcare professionals assisted parents in encouraging healthy behavior in the child by providing them information regarding diet, PA and SB strategies for promoting healthy behaviors before difficulties were encountered.

The Melbourne InFANT program is registered with the International Standard Randomized Controlled Trial Number Register (ISRCTN81847050). Ethical approval was granted by the Deakin University Ethics Committee [ID number: EC 175-2007 (21.07.2007); additional approval for present secondary analysis (13.01.2023)] and by the Victorian Office for Children (Ref: CDF/07/1138). De-identified data for this secondary analysis were made available on the 17th of April 2023. This study was reported according to Consolidated Standards of Reporting Trials (CONSORT) guidelines (29).

### Participant recruitment and randomization

Participants were recruited using a two-stage random sampling method between the 7th of April 2008 and the 12th of December 2008. In total, 14 out of the 28 local government areas (LGAs), located within a 60-km radius of Deakin University (Burwood, Victoria, Australia) were randomly ordered and selected [see protocol paper; (30)]. One eligible LGA council declined participation and was substituted with the next LGA on the randomly ordered list. The Census of Population and Housing: Socio-Economic Indexes for Areas area-level index of relative disadvantage, based on characteristics such as income, low-skill occupation, and number of people without qualifications (31), was used to divide these areas into three socio-economic categories for description purposes, but was not part of the randomization process (30). Three LGAs were classified as “low”, eight as “medium”, and three as “high” socio-economic status.

All LGAs provided free first-time parent groups as part of their universal health care that were formed and facilitated by the Maternal and Child Health service. Eligible first-time parent groups (62/103 groups) within participating LGAs were randomly selected and invited to participate in InFANT. For groups to be included in the study, they had to have a minimum of eight parents consenting to participate in writing, or a minimum of six in low socio-economic areas. Participating parents had to be proficient in the English language. If a parents' group declined or was ineligible, the next group on the randomly ordered list within the same LGA was selected and approached.

**Figure 1:**
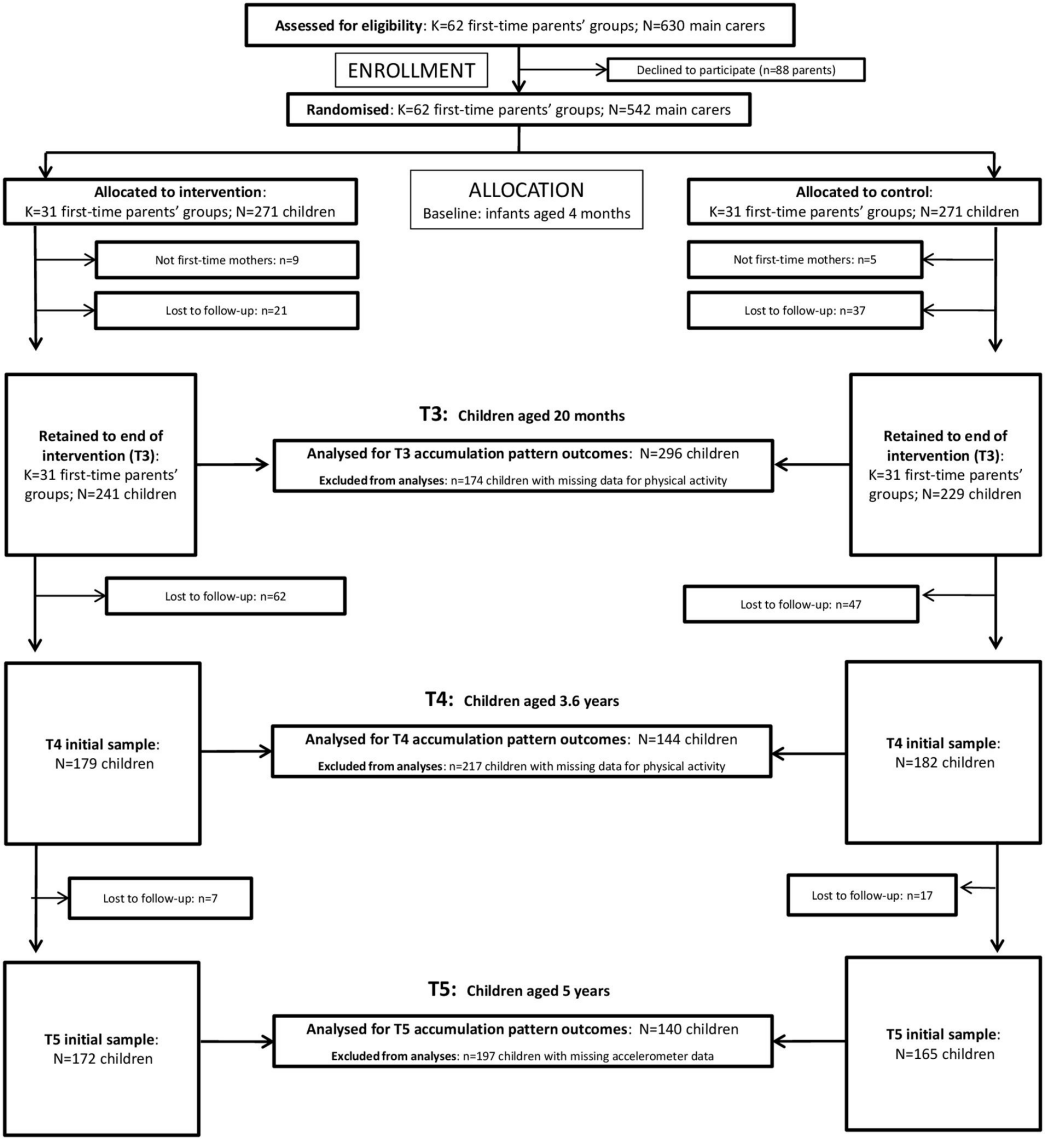
Flowchart of the InFANT study participants.

Eventually, 62 first-time parent groups (*n* = 542 participants out of the 630 eligible main carers; mean child age was 4 months) enrolled in the trial and were randomly assigned (stratified by LGA) to either the intervention (*n* = 271) or control group (*n* = 271; Figure 1). Despite welcoming parents of all genders, the participating parents were all mothers (in line with the predominant attendance of mothers with their babies in parent groups).

Randomization was performed by an independent statistician using a computer-generated sequence that was transferred into sealed sequential envelopes. After providing written consent but before participants had their first measurement session, they were informed of their group assignment. Due to the nature of the intervention, double blinding was not feasible. However, to minimize potential bias, study personnel responsible for data entry were blinded to the participant's group allocation throughout the study (30).

### Sample size estimation and justification

This study is a secondary analysis. Original sample size estimation and justification have been reported elsewhere (30), this suggested that estimated sample sizes of 394 and 315 at the two follow up data collections will allow us to detect meaningful differences between intervention and control groups at 3.5 and 5 years of age. From the allocated 542 participants, 480 parents attended their follow up measurement at 19 months (T3), 361 at 3.5 years (T4) and and 337 at 5 years of age (T5) (see Figure 1 for the participant flowchart).

### Intervention

The intervention program has been described in detail elsewhere (30). Briefly, it consisted of six 2-hour group sessions delivered by an experienced dietician quarterly over 15 months, in addition to their usual first-time parent sessions and individual care from their MCH nurse. The intervention was delivered within MCH nurse-led first-time parent groups using an anticipatory guidance framework (26) and a multimodal, interactive, format. Intervention components were described in more detail previously (32). The sessions included a short information video designed by the InFANT team with additional information provided by the registered dieticians from their handbook and included interactive activities and discussions. The sessions emphasized peer support, mutual respect, encouragement techniques, and shared problem-solving. The program, drawn on parenting support theory, focused on increasing parents' awareness of their influence on their children's behavior and improving their knowledge and skills related to eating habits, PA, and SB (28). While the intervention focused on increasing active play time and decreasing SB including screen time and time spent restrained, in line with national guidelines, some messages specifically focused on the accumulation of PA and SB. For example, strategies for breaking up long times in restraint positions such as encouraging their child to walk alongside the pram for short, building up to longer periods, rather than spending the whole time in the pram. The intervention sessions were conducted in community venues such as Maternal and Child Health centers (MCH) and local libraries. The control group received newsletters on general child health topics, such as common childhood illnesses, every three months for fifteen months. Of those participants in the intervention arm who completed the trial, 68% attended the majority of intervention sessions (4 or more of the 6 sessions) (33). The control group received the usual individual care from their MCH nurse and their first-time parent sessions.

### Data collection and measures

Measures were taken at baseline [T1 (2008): child age approximately 4 months], mid-intervention (T2: approximately 9 months), intervention conclusion [T3 (2010): approximately 19 months], and at two post-intervention follow-ups [T4 (2011): approximately 42 months and T5 (2013): approximately 60 months]. Accelerometer data were only collected at time points three, four and five, because most children were not able to walk at earlier time points. Consequently, the present secondary data analysis study focuses on data from time points T3–T5 only. Demographical data were collected at the first time point (baseline) and were used to describe the sample and for covariate adjustment.

#### Demographics

At T1, the parent was asked to report child date of birth and sex [binary (boy/girl)]. Child ages at T3, T4, and T5 were calculated based on the date of birth and date of testing. Parents also reported their highest level of education, which was dichotomized into “no university degree” and “university degree”.

#### Physical activity and sedentary time patterns

To assess PA and sedentary patterns at T3, T4 and T5, children wore an ActiGraph™ GT1M (Pensacola, USA) accelerometer on the right hip using an elasticized belt. Parents were provided with instructions on how to fit their children with the monitors and asked for their children to wear it for eight consecutive days during waking hours, except for during water activities. Data were considered valid when children had a minimum of four days of data with a wear duration of at least 7.4 h, as previous studies have demonstrated that this is sufficient to capture habitual PA in young children and yields reliable estimates (34). The 15-second accelerometer epoch data were processed using customized Excel macros.

Non-wear time was defined as twenty minutes of consecutive zeros. Accelerometer count data were classified as sedentary time (<100 counts per minute), light-intensity PA (LPA) (>101 and <1,680 counts per minute) and MVPA (>1,680 counts per minute) (35). To capture insights beyond total time and examine accumulation patterns, the duration and frequency of PA and sedentary bouts was determined based on the median bout length of the accelerometry data for this sample. First, to get a general idea of the preferred length for each intensity, the median bout length in bouts over 0.25 min (i.e., one 15-second epoch; minimum recorded duration) was calculated. Since all observed medians were under one minute (about 0.25 min for MVPA, 0.47 min for LPA, and 0.50 min for sedentary), bouts were classified as sustained periods lasting one minute or longer in each intensity. This is consistent with classifications used in this age group in other cohorts (36, 37). No interruptions in intensity within these bouts were allowed, based on previous recommendations for sedentary bouts (38) and in the absence of recommendations for PA bouts. Furthermore, the number of sedentary breaks was also determined, defined as the number of interruptions in sedentary time where counts exceeded 25 counts per 15s epoch (12, 39). All accelerometry variables were averaged across the valid days.

### Statistical analysis

Stata v.17.0 (StataCorp, College Station, TX, USA) was used to conduct all analyses. An analytical sample was created consisting of participants that had valid accelerometry at least one timepoint (as defined above) and covariates (age, sex, and highest level of education of the parent) data at baseline. All variables were checked for outliers, implausible values, and normality. All variables seemed appropriate, and no data transformations were executed. Descriptive analyses (mean and standard deviations) were used to describe sample characteristics.

Restricted maximum-likelihood multilevel modelling was used to assess the effect of the intervention on accumulation patterns at T3, T4 and T5 (*p* < 0.05). Given the nested and longitudinal nature of the data, a random intercept at the group level was included. Additionally, an unstructured residual correlation structure on the individual level was used for a more flexible approach and reduction of bias in the fixed effects. Intention-to-treat analyses were used for all analyses.

Two models (crude and adjusted) were fitted to estimate the intervention effects. The crude model included fixed effect terms to estimate intervention effects at each of the three time points and adjusted for time (continuous, months) and total wear time (continuous, min/day) only. Adjusted models additionally controlled for sex, the highest level of maternal education, and child age (40–42). As sex and maternal education level may have varying effects across time points, interaction terms between these covariates and time were included in the models. Employing the maximum likelihood approach to jointly estimate intervention effects across multiple time points allowed individuals with data at one or more time points to contribute to intervention effect estimation at all time points. This approach enhances efficiency and robustness in handling missing data compared to analyzing separate outcome points in distinct models.

Our study employed a RCT design specifically to mitigate potential baseline differences between intervention and control groups, considered the best way to investigate the effect of a new treatment (i.e., intervention effect) (43). By using the gold standard RCT design, it is assumed that all baseline differences between the groups are due to chance, and thus adjusting for baseline values is unnecessary (43). The intervention began when participants were four months old, a developmental stage where measurement of PA, as typically defined, is not possible. As such, it was not possible to collect baseline measures of the outcome in the present study. In effect, both groups were equivalent in terms of their PA at baseline as it had not yet begun, they had not yet become ambulatory. It is common practice in early intervention trials to not collect behavioral outcome measures at baseline (e.g., PA, diet) as these behaviors have not yet been established [e.g., (44, 45)]. Consequently, we did not adjust for baseline behaviors. Covariates were determined *a priori* based on literature; therefore, models were not compared using maximum log-likelihood. Time was integrated into the crude model as a continuous variable to adjust for the time-varying factors. The crude model was controlled for wear time, because it had a substantial influence on the outcomes that was more a result of a methodological factor than confounding. Adjusting for wear time would result in a more accurate estimation of the intervention effect in the crude model. The age of the child was integrated because preschoolers have different movement behaviors at different ages. A *p*-value of ≤0.05 was set as the level of statistical significance.

## Results

### Participant characteristics

A flow diagram including the number of included participants at each time-point is presented in Figure 1. In total, 480, 361 and 337 children were provided with accelerometers at T3, T4 and T5, respectively. Of these children, 296, 144 and 140, respectively, had valid accelerometry data. Baseline characteristics (mean ± SD) for the participants in the intervention and control group were presented separately. The mean age at T3 was 18.5 (ranging from 15.7 to 24.5) and 18.0 (ranging from 15.6 to 27.4) months for the intervention group and control group, respectively (Table 1). Both groups had an approximate half-half ratio of boys and girls. At T3, in the control group 66.9% of the parents contained a university degree in comparison to 51.7% the intervention group. Further sample characteristics have been previously reported (30).

**Table 1:** Characteristics of the InFANT program population with valid data at T3 (approximately 18 months; 2010).

Characteristics	Baseline (T3) (*n* = 296)	
	Intervention (*n* = 145)	Control (*n* = 151)
Age of child in months mean ± SD (range)	18.5 ± 2.0 (15.7–24.5)	18.0 ± 1.8 (15.6–27.4)
Sex of child *n, %*		
Boy	76 (52.4%)	79 (52.3%)
Girl	69 (47.6%)	72 (47.7%)
Highest level of education of the mother^a^ *n*, %		
No University	70 (48.3%)	50 (33.1%)
University	75 (51.7%)	101 (66.9%)

InFANT, The Melbourne Infant Feeding, Activity and Nutrition Trial; SD, standard deviation.

a The highest level of education of the mother variable was dichotomized (no university vs. university) from 6 original categories: no formal qualifications, year 10 or equivalent, year 12 or equivalent, trade/apprentices, certificate/diploma, university degree or higher university degree.

### Intervention effects on physical activity and sedentary patterns

Most time was spent in sedentary bouts (237.3–253.9 min), followed by LPA (110–121.4 min), and MVPA (11.3–30.5 min) as last. Over time (and adjusted for sex, the highest level of maternal education, and child age) there was an increase in the total number of sedentary breaks for both groups. No significant differences were observed between the intervention group and the control group in the time spent in sedentary, LPA and MVPA bouts lasting longer than 1 min at any time-point (see Table 2). However, the total number of sedentary breaks significantly differed between groups at T4 (both models) and T5 (adjusted model only). Specifically, the intervention group had more sedentary breaks per day on average than the control group at T4 [284.7 vs. 275.3 breaks/day; adj *β* (95%CI) = 10.9 (2.98, 18.9)] and T5 [300.9 vs. 288.9 breaks/day; adj *β* (95%CI) 7.94 (0.03, 15.86)].

**Table 2:** Intervention effects on activity patterns at T3 (2010; approximately 19 months), T4 (2011; 42 months), and T5 (2013; 60 months).

Activity patterns	Time point 3 (*n* = 296) Age range: 15.6–27.4 months				Time point 4 (*n* = 144) Age range: 39.0–51.0 months				Time point 5 (*n* = 140) Age range: 57.6–70.4 months			
	I *n* = 145 Mean (SD)	C *n* = 151 Mean (SD)	Crude^c^ *β* [95% CI]	Adjusted^d^ *β* [95% CI]	I *n* = 78 Mean (SD)	C *n* = 66 Mean (SD)	Crude^c^ *β* [95% CI]	Adjusted^d^ *β* [95% CI]	I *n* = 77 Mean (SD)	C *n* = 63 Mean (SD)	Crude^c^ *β* [95% CI])	Adjusted^d^ *β* [95% CI]
Sedentary bouts^a^	237.3 (46.3)	247.5 (63)	0.62 [−8.38;9.62]	−0.46 [−9.75;8.82]	246.8 (42.9)	251.9 (68.5)	−6.17 [−19.70;7.38]	−5.56 [−19.75;8.64]	253.2 (51.7)	253.9 (43.3)	−7.81 [−22.18;6.57]	−6.47 [−21.10;8.16]
LPA bouts^a^	121.4 (31.8)	125.6 (27.5)	−6.17 [−19.70;7.38]	−1.55 [−8.37;5.27]	124.6 (24.6)	120 (23.3)	5.24 [−2.16;12.63]	4.20 [−3.65;12.05]	115.3 (24.7)	110 (21.6)	4.69 [−2.31;11.86]	4.88 [−2.51;12.27]
MVPA bouts^a^	11.3 (7.3)	11.3 (5.8)	0.41 [−1.09;1.90]	0.66 [−0.85;2.18]	21.4 (11.8)	23.9 (12.8)	−1.82 [−5.57;1.92]	−2.25 [−6.06;1.57]	29.6 (12.4)	30.5 (14.4)	−0.78 [−4.96;3.40]	−1.89 [−6.00;2.21]
Sedentary breaks^b^	252.5 (38.0)	260.7 (40.5)	−0.50 [−7.11;6.11]	−0.05 [−7.09;6.99]	284.7 (33.0)	275.3 (27.7)	**10.59 [2.98;18.19]** *	**10.90 [2.98;18.90]** *	300.9 (31.0)	288.9 (33.7)	6.31 [−1.38;13.99]	**7.94 [0.03;15.86]** *

I, intervention; C, control; CI, confidence intervals; LPA, light-intensity physical activity; MVPA, moderate- to vigorous-intensity physical activity.

a Bouts were defined as continuous time in one intensity (sedentary time, LPA or MVPA) of at least one minute, without any interruptions in another intensity (in minutes).

b Sedentary breaks were defined as the number of interruptions in sedentary time where counts exceeded 25 counts per 15 s epoch (#).

c Estimated intervention effects [β (95% CI)] of the crude model adjusted for time and wear time only.

d Estimated intervention effects [β (95% CI)] of the adjusted model, additionally adjusted for age, sex, and highest educational level of the mother.

* Bold values denote statistical significance at the *p*-value of ≤0.05.

## Discussion

This study investigated whether the Melbourne InFANT program influenced preschoolers' PA and SB patterns, specifically examining the duration and frequency of sedentary, light-, and moderate- to vigorous-intensity activity bouts, as well as the number of sedentary breaks, to move beyond total time-based measures. Results suggest that there was no intervention effect on sedentary, LPA and MVPA bouts. However, the intervention had significant beneficial effects on the number of sedentary breaks, which were more pronounced at 2 and 3.5 years post intervention (approximately 3.6 and 5.0 years of age). More sedentary breaks were observed in the intervention than the control group. These findings are important because they demonstrate the possibility of modifying accumulation patterns in children under five years of age to benefit short- and long-term health, given these impacts persisted beyond the end of the intervention.

To our knowledge, this is the first study to investigate the effect of a parent-based intervention on accumulation patterns, defined as bouts and breaks in PA and sedentary time, in children aged 0–5 years. Previous studies in older children have investigated the possibility of modifying accumulation patterns. While older children typically have different (less sporadic) activity patterns than younger children (46), which complicates comparison, there are some similarities in findings. For example, the study of Verswijveren et al. (19) did not show an effect on PA and sedentary patterns at conclusion of a home- and school-based PA and SB intervention (Transform-Us!). However, longer term follow-up showed a significant increase in sedentary breaks for the groups that received an intervention in comparison to the control group at 30 months after the initiation of the intervention (20). An explanation for this finding in the current study could be that the program contained messages that focused on reducing restrained behavior (i.e., prolonged sitting), whereas it did not specifically focus on PA and sedentary bouts, rather just on increasing time spent active and reducing time sedentary. This suggests that intervention messages may need to be specifically targeted to changing activity patterns to be effective. It is also possible that interventions may be most effective if focused on changing sedentary breaks rather than on changing PA patterns. However, with such a limited amount of evidence available on interventions to change activity patterns, our understanding of these impacts remains speculative.

One explanation for insignificant and small results for PA and sedentary bouts could be that the PA component was undermined by the nutritional component of the Melbourne InFANT program. Furthermore, additional investigations into behavior change mechanisms in the InFANT study revealed that parents of young children generally assume that their children are naturally inclined to be active (47), and thus it's reasonable to assume that parents may have been less open to messages aimed to increase their child's PA (active play). Let alone to increase PA and sedentary bouts.

Another explanation for the lack of significant effects from the parent-based Melbourne InFANT program on the PA and sedentary bouts could be the influence of the out-of-home environment. According to the Australian Bureau of Statistics, approximately 93% of all children aged 3–5 went to either preschool or kindergarten in 2010 (48, 49). Considering that the intervention primarily targeted parents rather than movement behavior in ECEC settings, it is likely that movement captured during ECEC attendance was not directly impacted by potentially improved parenting skills around movement behavior in the intervention group vs. the control group. Moreover, several studies found significant differences in PA levels for preschoolers at ECEC compared to home care, where preschoolers at ECEC centers had higher levels of PA (29). Thus, changes in parental skills may not have been reflected in assessing whole-of day measurements. Using a whole-of-day, hybrid strategy where interventions target both parents and out-of-home settings could therefore help us understand how to change these patterns.

### Limitations and strengths

Firstly, the present secondary data analysis was, to the best of the authors' knowledge, the first to assess the impacts on PA and sedentary patterns at this young age group. Secondly, the accelerometers used for measuring movement patterns are considered valid and reliable instruments to measure movements in this age group (50). Thirdly, advanced analytical models were used to appropriately deal with missing cases, allowing for the analysis of multiple time points (51).

Despite its strengths, this study has some limitations as well. Firstly, accelerometers have difficulties in making a distinction between sedentary time and low LPA standing since these are both stationary behaviors. Consequently, this measuring method could have underestimated LPA and overestimated sedentary time (52). In addition, these devices do not capture data on setting and/or context of the movement behaviors. For example, a day at home would result in different accumulation patterns than a day at ECEC. More broadly, several contextual factors that may influence children's PA and SB were not measured, such as availability of screens and play spaces in the home environment, and parental PA and role modelling. These unmeasured factors may have contributed to intervention responsiveness. We also did not examine whether session attendance or intervention exposure was associated with the outcomes reported in this paper. Secondly, the previous studies on the Melbourne InFANT program showed that children with parents with lower educational levels did benefit more from the intervention (53). Therefore, higher attrition rates at the follow-ups for children whose parents had lower educational levels could have undermined the intervention effect. This also makes the results less generalizable. Despite the use of retention strategies (e.g., Christmas cards), there was a significant loss-to follow-up of participants between T3 and T4, mostly due to loss of contact with the parents. Lastly, while the RCT design minimized baseline differences between groups, the lack of baseline accelerometer data limits causal inference regarding individual-level change over time. This was unavoidable, as the intervention commenced at four months of age, prior to the establishment of ambulatory movement behaviors.

## Conclusions

This study was to our knowledge the first to examine the effect of a parent-based intervention on accumulation patterns of PA and sedentary time in children aged 0–5 years. A beneficial intervention effect was observed for the number of sedentary breaks at approximately 3.5 and five years of age, however, no significant effects were identified for PA and sedentary bouts. These findings are important because they demonstrate the possibility of modifying accumulation patterns in children under five years of age to benefit short- and long-term health. However, accumulation patterns may be hard to change due to the sporadic nature of movement in children, especially at younger ages. Moreover, sedentary breaks could potentially be conceptually different than PA and sedentary bouts. Future studies should aim to compare the efficacy of parent-based interventions to other approaches such as out-of-home or hybrid interventions, investigate the effectiveness of intervention messages focusing on accumulation patterns instead of PA in general, and explore the feasibility of modifying activity accumulation patterns in the under-five population.

## Data Availability

The data analyzed in this study is subject to the following licenses/restrictions: the data underlying this study cannot be publicly shared as it was not collected for open access purposes. Requests to access these datasets should be directed to Prof Kylie Hesketh, kylie.hesketh@deakin.edu.au.
